# Four novel *Planctomicrobium* species isolated from sewage sludge or leakage water of a compost heap in Northern Germany

**DOI:** 10.1038/s41598-026-36544-9

**Published:** 2026-01-30

**Authors:** Nicolai Kallscheuer, Gaurav Kumar, Jonathan Hammer, Tom Haufschild, Christian Jogler

**Affiliations:** 1 Department of Microbial Interactions, Institute of Microbiology, Friedrich Schiller University, Jena, Germany; 2 Cluster of Excellence Balance of the Microverse, Friedrich Schiller University, Jena, Germany

**Keywords:** *Planctomycetota*, Limnic strains, Schlesner strain collection, *Planctomicrobium piriforme*, *Planctomycetaceae*, Microbiology, Molecular biology

## Abstract

Four non-pigmented, off-white to ivory strains, SH527^T^, SH661^T^, SH664^T^ and SH668^T^, have been isolated from sewage sludge or leakage water of a compost heap in Northern Germany as part of an isolation campaign conducted by Heinz Schlesner, Kiel University in the 1980s-early 2000s. Analyses based on five phylogenetic markers place the novel strains in the genus *Planctomicrobium* in the family *Planctomycetaceae* within the phylum *Planctomycetota*. All four strains are aerobic heterotrophs with a neutrophilic and mesophilic growth profile. Genome sequencing revealed relatively small genomes compared to the relatives in the same family, with strain SH668^T^ having the currently smallest genome (4.81 Mbp) of any described member of the family *Planctomycetaceae*. The analysis of genome-encoded features suggests a metabolism better adapted to the degradation of polysaccharides rather than of lignocellulose-derived aromatic compounds. The collected data justifies the delineation of the novel isolates from the sole species in the genus, *Planctomicrobium piriforme.* The four isolates belong to four separate novel species, for which we introduce the names *Planctomicrobium limosum* sp. nov., *Planctomicrobium stercoris* sp. nov., *Planctomicrobium aquicomposti* sp. nov. and *Planctomicrobium mucosum* sp. nov. The novel taxa are represented by the following isolates as respective type strains: SH527^T^ (= CECT 30937^T^ = KCTC 102136^T^), SH661^T^ (= DSM 116760^T^ = KCTC 102128^T^), SH664^T^ (= DSM 116388^T^ = KCTC 102081^T^) and SH668^T^ (= DSM 116585^T^ = KCTC 102080^T^).

## Introduction

The phylum *Planctomycetota* represents a ubiquitous and ecologically significant group of bacteria with uncommon cell biological features and complex life cycles^1^. Their cell plan resembles that of Gram-negative bacteria, but with several variations^2^: extensive membrane invaginations, condensed DNA and asymmetric cell division by budding^3,4^. In some species, yet uncharacterized conspicuous subcellular structures exist^5^. Planctomycetes contain peptidoglycan cell walls^3,4^; however, many of the otherwise essential bacterial genes involved in peptidoglycan biosynthesis have been shown to be dispensable as exemplified in the limnic model species *Planctopirus limnophila*^6^. The Z ring assembly protein FtsZ, the hallmark protein for cell division by binary fission, is also absent in planctomycetes^6,7^.

Members of the phylum *Planctomycetota* thrive in marine, freshwater and terrestrial environments, where they significantly influence biogeochemical cycles^8–10^. Their role in the nitrogen cycle is particularly notable, with bacteria in the class “*Candidatus* Brocadiia” contributing 30–50% of nitrogen loss in oxygen-limited marine ecosystems through anaerobic ammonium oxidation (anammox)^11,12^. Members in all described classes contribute to carbon cycling by degrading complex carbohydrates, including decorated polysaccharides, using a yet uncharacterized uptake system and an array of carbohydrate-active enzymes (CAZymes)^13–15^. This heterotrophic lifestyle is in line with the frequent isolation of planctomycetes from biotic surfaces of eukaryotic hosts reported in numerous studies. These include sea grasses^16,17^, green algae^1,18^, red algae^19,20^, sponges^21,22^ or jellyfish^23^ where they can account for up to 80% of the bacterial community^1,17^. Planctomycetal bacteria are frequently associated during cyanobacterial blooms as well^24,25^ and attach to biotic^26^ and abiotic surfaces if submerged^27,28^. Surprisingly, while mostly mesophilic, several planctomycetal strains have been isolated from submarine volcanic sites^29,30^.

Several studies have identified characterized phylum members as producers of bioactive molecules^31^. While their role as untapped source of bioactive compounds has for long only been assessed by genome mining and confirmed activities in cell extracts^32,33^, the first chemical structures have been unveiled in the last five years^34–37^. Investigation of their cell biology and the biotechnologically exploitable potential is guided by the construction of genetic tools^38,39^, allowing the study of individual gene functions through deletion mutants or by the introduction of heterologous genes encoding reporter proteins.

Despite their significance, the diversity of *Planctomycetota* remains underexplored, with many novel lineages awaiting discovery and characterization^40^. The current open collection of axenic cultures includes ca. 140 strains. In this study, we contribute four novel species of the genus *Planctomicrobium* for which the type strains have been isolated from sewage sludge and leakage water of a compost heap. The genus belongs to the family *Planctomycetaceae*, the first described family of the class *Planctomycetia*, and currently contains only one described species, *Planctomicrobium piriforme*. The type strain P3^T^ was isolated from a boreal peat bog in Russia^41^. The here presented isolates are part of the strain collection of Heinz Schlesner (“SH” strains, based on his initials), a pioneer in the research on budding bacteria including planctomycetes at Kiel University. He isolated several hundreds of novel strains from various locations in Northern Germany but also from international collaborations in the last two decades of the 20th century^42^. In addition to his strain collection, he also contributed to the characterization of many of the first described members of the phylum, e.g. *Pirellula staleyi*^43^, *Blastopirellula marina* (type strain SH106^T^)^44^ and *Rubinisphaera brasiliensis* (type strain SH180^T^)^45^ as well as of the first planctomycete with a sequenced genome, *Rhodopirellula baltica* (type strain SH1^T^)^46^. For the above-mentioned novel strains clustering next to *P. piriforme* P3^T^, we performed the characterization of phenotypic, genomic and genome-encoded features. Since classical chemotaxomomic methods turned out to have insufficient resolution for previously described closely related strains belonging to the family *Planctomycetaceae*^22^, these analyses have been omitted for the here described isolates. Instead, the phylogenetic inference is based on established single gene- or whole genome-based phylogenetic markers and well-established threshold values used for the phylum.

## Materials and methods

### Sampling, isolation of strains and 16S rRNA gene sequencing

Strain SH527^T^ was isolated from sewage sludge, whereas the three strains SH661^T^, SH664^T^ and SH668^T^ were isolated from leakage water of a compost heap in Northern Germany. Details on the isolation are described in published studies supervised by Heinz Schlesner^47,48^. The following media were used for cultivation of the strains: M30PY (SH527^T^) or M1aPY (SH661^T^, SH664^T^ and SH668^T^). Media were prepared as previously described^49^. The addition “PY” indicates that the media contained 0.25 g/L peptone and 0.25 g/L yeast extract. All strains grew on plates solidified with 15 g/L agar or in liquid medium. The 16S rRNA gene of the isolates was amplified by PCR and purified based on a standardized workflow^50^ and was sequenced at Macrogen Europe (Amsterdam, The Netherlands).

### Physiological analyses

For determination of the temperature optimum for growth, 100 µL supernatant of an exponentially growing culture was plated on the respective media mentioned above. Plates were incubated in triplicates at temperatures ranging from 4 to 42 °C as previously described^51^. The temperature at which colonies appeared the earliest was considered the temperature optimum for growth. The pH optimum for growth was determined in liquid cultures with 100 mM of the following buffering agents: 2-(*N*-morpholino)ethanesulfonic acid (MES) for pH 5.0 and 6.0, 4-(2-hydroxyethyl)-1-piperazineethanesulfonic acid (HEPES) for pH 7.0, 7.5 and 8.0 or *N*-cyclohexyl-2-aminoethanesulfonic acid (CHES) for pH 9.0 and 10.0. Growth was evaluated by measuring the optical density at 600 nm (OD_600_). The experiment was conducted at 24 or 28 °C (the determined optimal temperature for each strain) in a BioTek Epoch2 microplate spectrophotometer (Agilent) with constant shaking over a cultivation time of two weeks. One measurement cycle lasted 30 min in total and consisted of two shaking periods interrupted by the measurement of the whole 96-well plate (Brand Plate pureGrade^™^ S, transparent sterile 96-well plates). To prevent condensation on the lid a temperature gradient was set up: the liquid was heated to 24–28 °C depending on the temperature optimum of the strains and the lid temperature was maintained at 2 °C above the incubation temperature. Obtained data points (from duplicate measurements) were corrected by subtraction of the OD_600_ of the medium blanks. The growth rate at each tested pH value was obtained from the maximal slope after plotting the ln(OD_600_) against the cultivation time.

### Microscopy and cell size determination

Light microscopy was conducted as previously described^51^. Briefly, cells from liquid cultures at the mid-exponential phase were mounted on a 1% agarose cushion (w/v, dissolved in dH_2_O). After the culture dried on the agarose cushion, a cover slip was placed on top and the edges were fixed with VLAP (33% vaseline, 33% lanoline, 33% paraffin, w/w) to prevent the cover slip from moving. Cells were imaged in an inverse Nikon Ti2 microscope equipped with a Nikon Plan Apo λ 100x immersion oil objective with a phase ring for phase contrast (PhC) images and without a phase ring for differential interference contrast (DIC) images, a Nikon DS-Ri2 camera, and the NIS-Elements software (version 5.30). Three-channel RGB images were transferred to FIJI^52^ to create a single-channel RGB image. Tiff files were transferred to BacStalk^53^ and cells were segmented using 25 and 15 pixels for cell size and minimum cell size, respectively. In total, three replicates with 150 cells each were analyzed. For data visualization, the obtained data was uploaded to SuperPlotsOfData^54^. For the creation of images visualizing cells, the brightness and contrast of PhC and DIC images were adjusted manually.

### Genomic DNA isolation, genome sequencing, annotation and analysis

Isolation of genomic DNA as well as genome sequencing, assembly and polishing were performed as previously described^51^ with the following modifications: For nanopore long-read sequencing the Native barcoding Kit 24 V14 (SQK-NBD114.24) was used with some alterations of the manufacturer’s protocol: The DNA control sample was omitted in most cases and the DNA sample volume was increased to 12 µL. For DNA repair and end-prep the incubation times were increased to 20 min at 20 °C and 20 min at 65 °C and the incubation time on the HULA mixer as well as the incubation time after adding nuclease-free water to the beads was increased to 10 min each. The incubation times for native barcode ligation and adapter ligation were increased to 30 min. The incubation time after adding elution buffer to the beads was increased to 15 min. Bovine Serum Albumin (Invitrogen) was added to the flow cell priming mix as recommended. The final libraries were loaded onto a primed R10.4.1 flowcell (FLO-MIN114, Oxford Nanopore Technologies). For strains SH664^T^ and SH668^T^ basecalling was performed using Dorado version 0.3.0 (Oxford Nanopore Technologies) with the basecalling model dna_10.4.1_e8.2_400bps_sup@v4.2.0, demultiplexing was performed using Guppy version 6.5.7 with the optional flag “–trim_barcodes” (Oxford Nanopore Technologies). For strains SH527^T^ and SH661^T^ basecalling, demultiplexing and filtering of reads with a Phred quality score ≥ 10 were performed with Dorado version 0.5.0 and 0.5.3 (Oxford Nanopore Technologies), respectively, with the basecalling model dna_10.4.1_e8.2_400bps_sup@v4.3.0. Quality control of the nanopore reads was performed with NanoPlot version 1.41.0^55^. The assembly was performed with Flye version 2.9.1 with the flag “--nano-hq” for the genomes of strains SH527^T^, SH664^T^ and SH668^[T56,57^. Flye version 2.9.3 with the flags “--nano-hq” and “--min-overlap 5000” was used for the genome assembly of strain SH661^T^. Medaka version 1.7.2 (Oxford Nanopore Technologies) was used for polishing of the raw assembly with the original long reads. Illumina sequencing for polishing with short reads was performed by Eurofins Genomics (Ebersberg, Germany). The final genome sequences were checked for completeness using Busco v5.8.2^58–60^. Coding density and DNA G + C content were analyzed with checkM v1.2.3. After annotation with prokka v1.14.6, the chromosome was rotated to the start codon of the replication initiator protein-encoding gene *dnaA* and was re-annotated with PGAP version 2025-05-06.build7983.

### Nucleotide sequence accession numbers

The 16S rRNA gene sequences were deposited in the GenBank database under the following accession numbers: PV904163 (SH527^T^), PV904164 (SH661^T^), PV904179 (SH664^T^) and PV904181 (SH668^T^). Genome sequence information is available from NCBI under the accession numbers CP195790 (SH527^T^), CP195791 (SH661^T^), CP195792 (SH664^T^) and CP195793 (SH668^T^).

### Phylogenetic and genome-based analyses

16S rRNA gene sequences of the novel isolates were extracted from the prokka-annotated genomes and were used for the identification of the current closest relatives using NCBI BLAST. Maximum-likelihood phylogenetic trees based on 16S rRNA gene sequences and multi-locus sequence analysis (MLSA) were computed for the novel strains and the described type strains of all species in the current phylum *Planctomycetota* (the MLSA-based tree only included members of the order *Planctomycetales* and the outgroup). The 16S rRNA gene sequences of *Opitutus terrae* (NCBI acc. no. AJ229235), *Kiritimatiella glycovorans* (acc. no. NR_146840) and *Lentisphaera araneosa* (accession number NR_027571) (members of the *Planctomycetota-Verrucomicrobiota-Chlamydiota* (PVC) superphylum outside of the phylum *Planctomycetota*) served as outgroup. Sequence alignments were performed with ClustalW^61^ and FastTree v2.2 was used for tree reconstruction with 1000 bootstrap replications^62^. The MLSA-based phylogeny was computed with autoMLST (automlst-simplified-wrapper tool) with 500 bootstrap replicates^63^ and the genomes of *Rhodopirellula baltica* SH1^T^ (GenBank acc. no. BX119912.1), *Pirellula staleyi* DSM 6068^T^ (acc. no. CP001848.1) and *Blastopirellula marina* DSM 3645^T^ (acc. no. GCA_000153105.1) (all belonging to the family *Pirellulaceae*) as outgroup. Phylogenetic trees were visualized with iTOL v6^64^. The 16S rRNA gene sequence similarity matrix was obtained with TaxonDC^65^ based on the ClustalW alignment that was also used for the construction of the phylogenetic tree. Average amino acid identities (AAI) and average nucleotide identities (ANI) were calculated using respective scripts of the enveomics collection^66^. Additional phylogenetic markers, i.e. *rpoB* sequence similarity and percentage of conserved proteins (POCP), were calculated as described^67,68^. The pangenome of selected strains was generated with anvi’o v.8^69^. Biosynthetic gene clusters (BGCs) were predicted with antiSMASH v.8.0^70^ and carbohydrate-active enzymes (CAZymes) with dbCAN3^71^.

## Results and discussion

### Phylogenetic inference

**Fig. 1:**
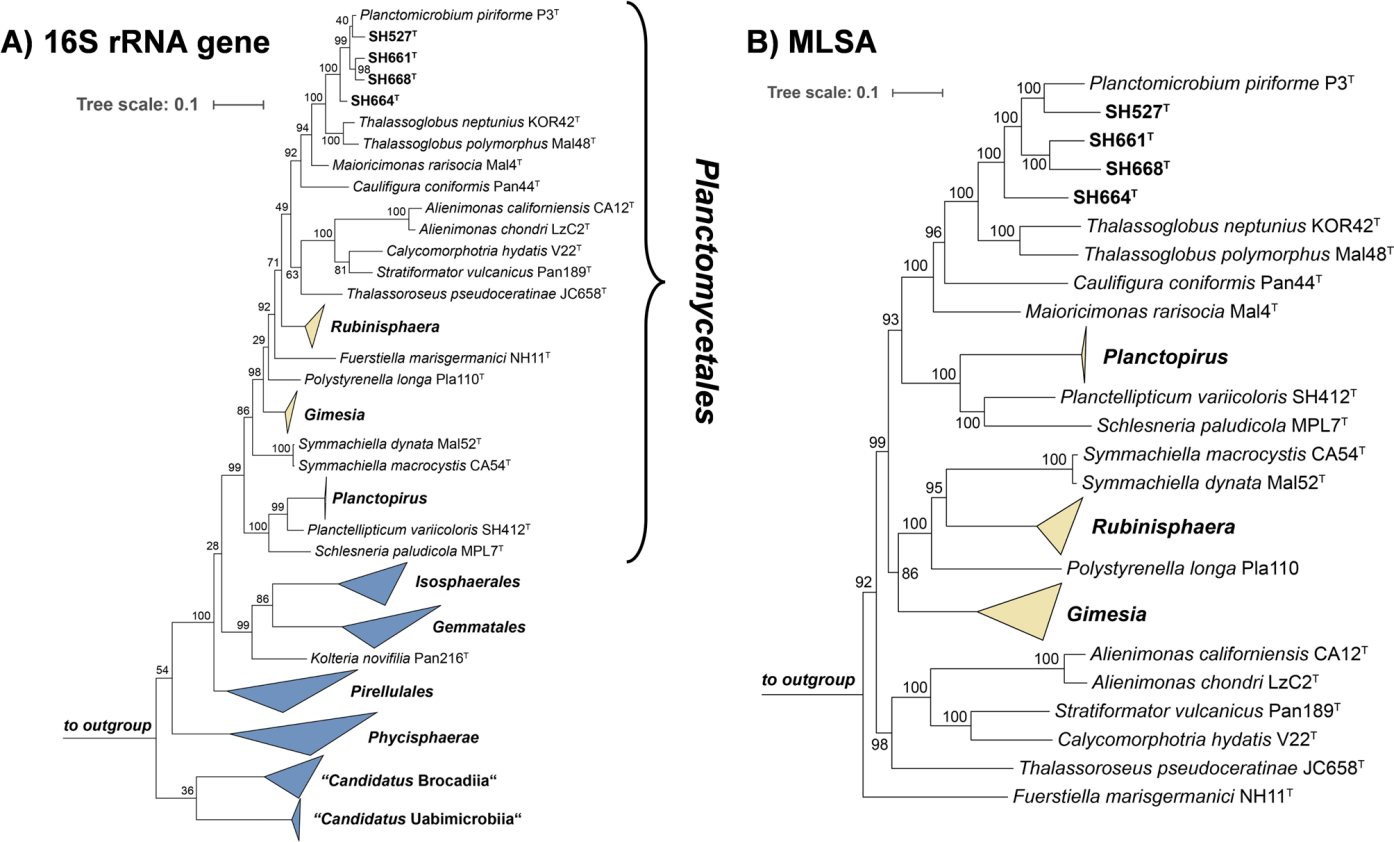
Phylogenetic placement. (**A**) Maximum likelihood phylogenetic tree based on 16S rRNA gene sequences showing the phylogenetic relationship of the novel isolates and the sole species of the genus *Planctomicrobium*. Bar, 0.1 substitutions per nucleotide position. (**B**) Multi-locus sequence analysis (MLSA)-based phylogenetic tree constructed with the genomes of characterized members in the order *Planctomycetales* (that currently includes *Planctomycetaceae* as the sole family). The tree was computed based on a set of at least 30 single-copy gene-encoding proteins in a maximum likelihood approach with 500 bootstrap replications. Bar, 0.1 substitution per amino acid position. Bootstrap values for both trees are given at the nodes (in %). Phylogenetic trees were visualized with iTOL v6.

**Fig. 2:**
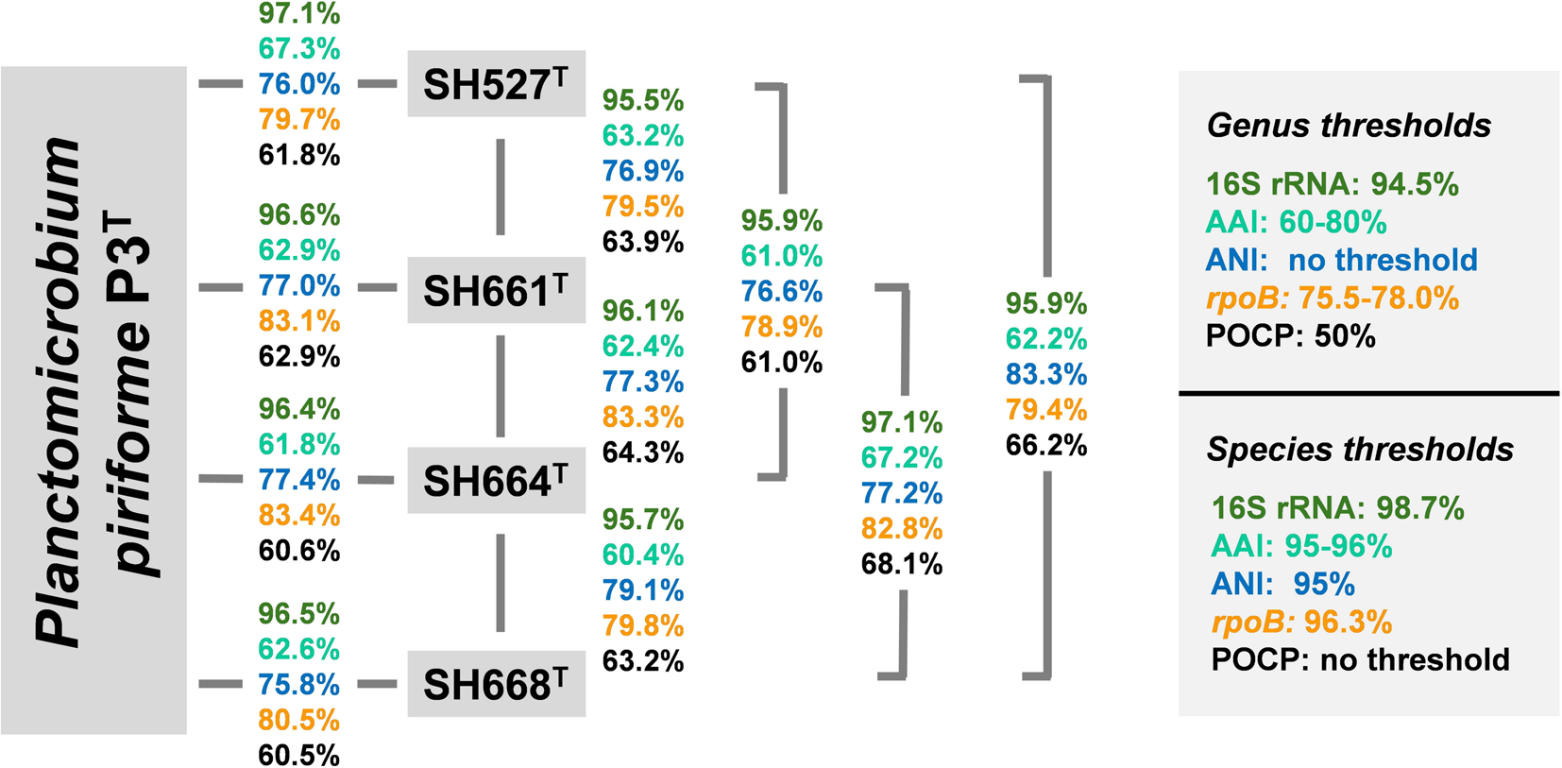
Comparison of phylogenetic markers for species delineation. Markers used: 16S rRNA gene sequence identity (16S rRNA), average amino acid identity (AAI), average nucleotide identity (ANI), sequence similarity of a partial sequence of the *rpoB* gene *(rpoB)*, percentage of conserved proteins (POCP).

Initial nucleotide blast analyses yielded maximal 16S rRNA sequence similarities in the range of 96–97% for the comparison of the novel isolates with the type strain P3^T^ of the species *P. piriforme* (family *Planctomycetaceae*)^41^. The high sequence similarity was also reflected in the clustering pattern in both phylogenetic trees (Fig. 1), indicating a relationship at least on the level of the same genus. None of the 16S rRNA sequence similarity values obtained during an all-vs-all comparison of the novel isolates and strain *P. piriforme* P3^T^ exceeded the species threshold of 98.7%^72^ (Fig. 2). This indicated that the four strains belong to four novel species of the genus *Planctomicrobium*. For getting further support on the phylogenetic status of the four isolates, additional markers and respective threshold values used for the delineation of genera and species were taken into consideration (Fig. 2). These included AAI, ANI, sequence similarity of a partial sequence of the gene *rpoB* encoding the β-subunit of RNA polymerase as well as POCP. Indeed, all markers applicable for species delineation yielded values below the threshold for all possible strain combinations analysed (Fig. 2)^67,73,74^. POCP values exceeding the genus threshold of 50% thereby ensure the relationship on the level of the same genus, *Planctomicrobium*^68^.

### Comparison of genomic features

Compared to *P. piriforme* P3^T^ (6.32 Mbp, non-closed draft genome), the genomes of the four novel isolates are significantly smaller (Table 1). The genome of strain SH668^T^ even has a size of only 4.81 Mbp and with that the currently smallest genome of any characterized member of the order *Planctomycetales.* Until now, this position was taken by *Calycomorphotria hydatis* V22^T^ with a genome size of 5.16 Mbp^5^. None of the compared strains has extrachromosomal elements. The DNA G + C content of the compared strains covers a range of 52–59% with a current gap between 53 and 57%. Regarding numbers of (protein-coding) genes per Mbp, relative numbers of hypothetical protein-encoding genes, coding densities and numbers of tRNA genes, the compared genomes only show minor differences (Table 1). The copy numbers of 5S, 16S and 23S rRNA genes vary from 1 to 3 with no obvious pattern. The number of copies of the 16S rRNA genes is always equal or higher compared to the number of copies of 5S and 23S rRNA genes in all strains including *P. piriforme* P3^T^. Strain SH527^T^ has a set of two directly adjacent 16S rRNA genes and the same organization for two adjacent sets of 5S–23S rRNA genes encoded distantly from the 16S rRNA genes. These are probably the result of gene duplication events explaining the higher total number of rRNA genes in this strain. A set of two 23S–5S rRNA genes interrupted by three open reading frames can also be found in the genome of strain SH668^T^.

**Table 1:** Comparison of genomic features.

Characteristics	*P*. piriforme P3^T^	SH527^T^	SH661^T^	SH664^T^	SH668^T^
Genomic features					
Genome size (bp)	6,317,004	5,492,586	5,859,625	5,215,884	4,807,360
Contigs	41	1 (closed)	1 (closed)	1 (closed)	1 (closed)
Plasmids	Inconclusive	No	No	No	No
DNA G + C content (%)	58.8	52.9	57.2	59.2	52.7
Genes	5016	4152	4752	4019	3813
Genes/Mbp	794	756	811	771	793
Protein-coding genes	4935	4060	4663	3924	3744
Protein-coding genes/Mbp	781	739	796	752	779
Hypothetical proteins*	1307	1010	1183	904	914
Hypothetical proteins (%)	26.5	24.9	25.4	23.0	24.4
Coding density (%)	85.7	83.1	85.8	84.7	85.4
rRNA genes (5S–16S–23S)	1,3,1	3,3,3	1,2,1	1,2,1	2,2,2
tRNA genes	49	43	44	48	43
Secondary metabolite-associated biosynthetic gene clusters (BGCs)					
Terpene	5	5	4	5	5
Type I polyketide synthase	1	1	1	1	1
Type III polyketide synthase	1	0	1	0	1
Ribosomally synthesized and post-translationally modified peptide product (RiPP)	2	1	1	1	1
BGCs per Mbp	1.4	1.3	1.2	1.3	1.7
Carbohydrate-active enzymes					
Glycoside hydrolases	50	33	46	39	31
Glycosyltransferases	53	48	42	42	37
Polysaccharide lyases	2	1	0	2	0
Carbohydrate esterases	15	20	21	21	14
Carbohydrate-bind. modules	13	5	11	7	10
Auxiliary activities	4	2	1	0	2
CAZyme genes (total)	137	109	121	111	94
CAZyme genes per Mbp	22	20	21	21	20

*Based on the PGAP-annotated genomes

### Construction of the pangenome and analysis of genome-encoded functionalities

**Fig. 3:**
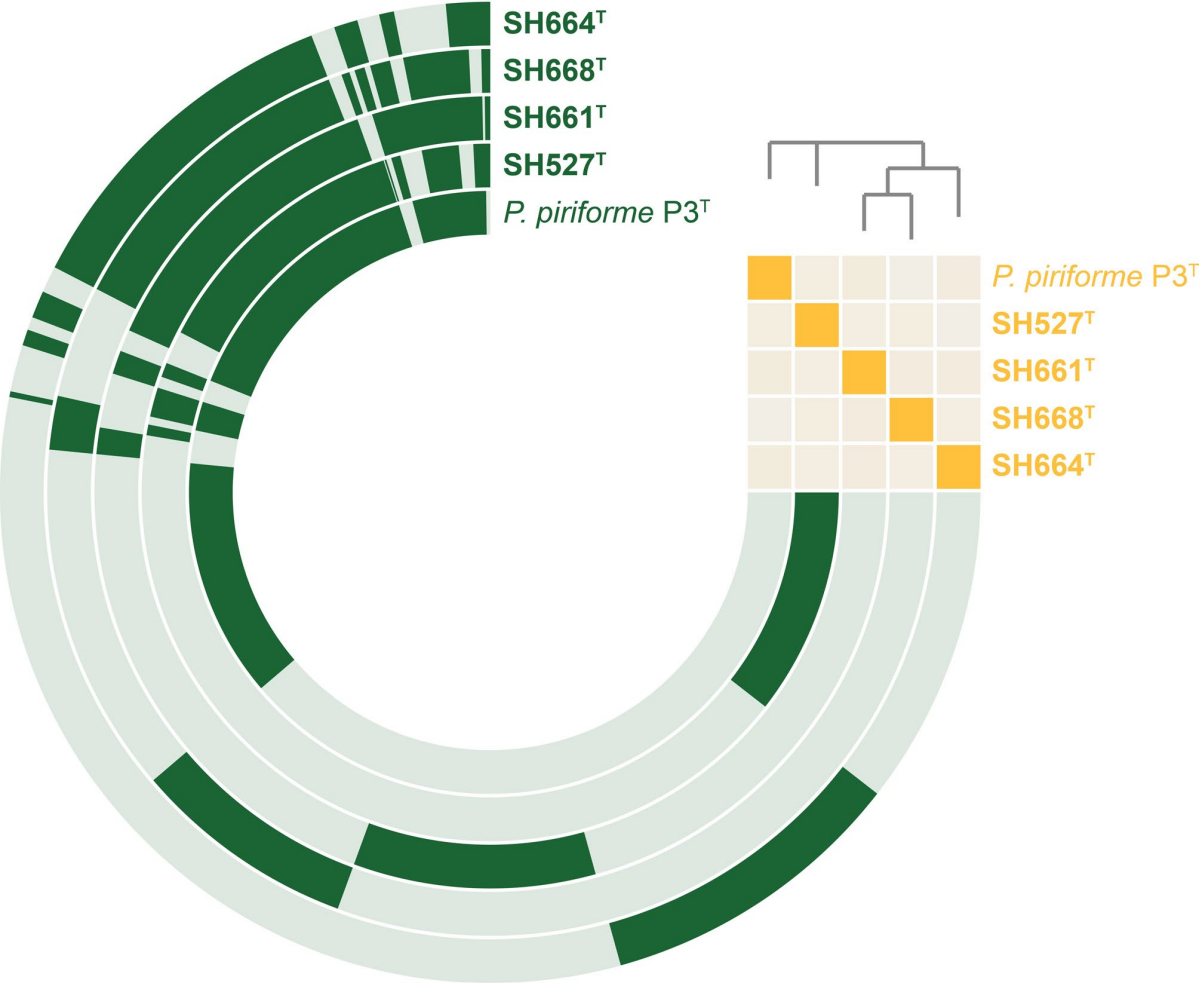
Pangenome reconstruction. Each open circle represents the pangenome of all genomes but is colored darker when the gene is present in the respective genome. The heatmap in the upper right corner indicates the degree of relationship based on ANI values (ANI ≤ 70%, pale orange to ANI = 100%, bright orange).

For a visualization of the genome-based similarity of the compared strains, a pangenome was constructed. The obtained *Planctomicrobium* pangenome consisted of 10,941 gene clusters, of which 1,669 are conserved in all five strains (core genome) (Fig. 3). The other genes are either singletons (strain-specific genes) or not conserved in all strains. The gene content of the singleton and core genomes sets was not analyzed in further detail in this study. However, in particular small genomes, as that of strain SH668^T^ with a suspected low number of accessory functions, are crucial to narrow down the list of conserved but uncharacterized genes in future studies. Such small genomes will help to study the functions of essential genes in the future, e.g. those involved in the asymmetric cell division by budding performed by members in various families within the phylum *Planctomycetota*.

The automated genome mining with antiSMASH yielded 7–9 biosynthetic gene clusters (BGCs) with potential relevance for secondary metabolite biosynthesis (Table 1). Four to five of these are probably involved in the biosynthesis of terpenoids or their precursors. Other predicted BGCs harbor putative type I or type III polyketide synthase-encoding genes or are involved in the synthesis of ribosomally-synthesized peptide products. The numbers of BGCs per Mbp are in the expected range for planctomycetal genomes of approximately one BGC per Mbp^31^; only for strain SH668^T^ the value is slightly increased due to its smaller genome size. The search for CAZyme-encoding genes yielded 90–140 hits per genome with similar numbers of hits per genome in the classes of glycoside hydrolases and glycosyltransferases (Table 1). The numbers of genes encoding carbohydrate esterase or proteins with auxiliary activities fell below five per genome for all five analyzed genomes.

Previously reported chitinolytic activity in *P. piriforme* P3^T^ could be traced back to the presence of a chitinase belonging to glycoside hydrolase family 18 (NCBI acc. no. SFI04223.1)^75^. Homologues with a protein sequence identity of 62%, 52% and 49% are encoded in the genomes of strains SH527^T^, SH661^T^ and SH668^T^, respectively. The protein appears to be absent in strain SH664^T^ indicating a lack of chitinolytic activity in this strain.

All five strains were isolated from terrestrial environments and more precisely from different forms of soil (peat bogs, compost) or wastewater. Hence, in addition to the analysis for CAZyme-encoding genes, the genomes were also inspected for genes coding for enzymes required for the aerobic degradation of lignocellulose-derived aromatic compounds (e.g. phenylpropanoids or hydroxybenzoic acids). The most common pathways include the β-ketoadipate pathway (*pca* and *cat* genes) and the gentisate pathway (*nag* genes) that include enzyme-catalyzed steps for the dioxygen-dependent fission of phenolics compounds and conversion of the linearized compound to intermediates of the tricarboxylic acid cycle^76^. Except for a single gene encoding a protein with similarity to the β-subunit of the protocatechuate 3,4-dioxygenase (PcaH), no other genes involved in the catabolism of aromatic compounds are encoded in the genomes of the five compared strains. This is a hint that the degradation of polysaccharides is the favored source of carbon and energy and not the carbon derived from the lignocellulose fraction of the rotting (plant) material.

### Phenotypic characterization

**Fig. 4:**
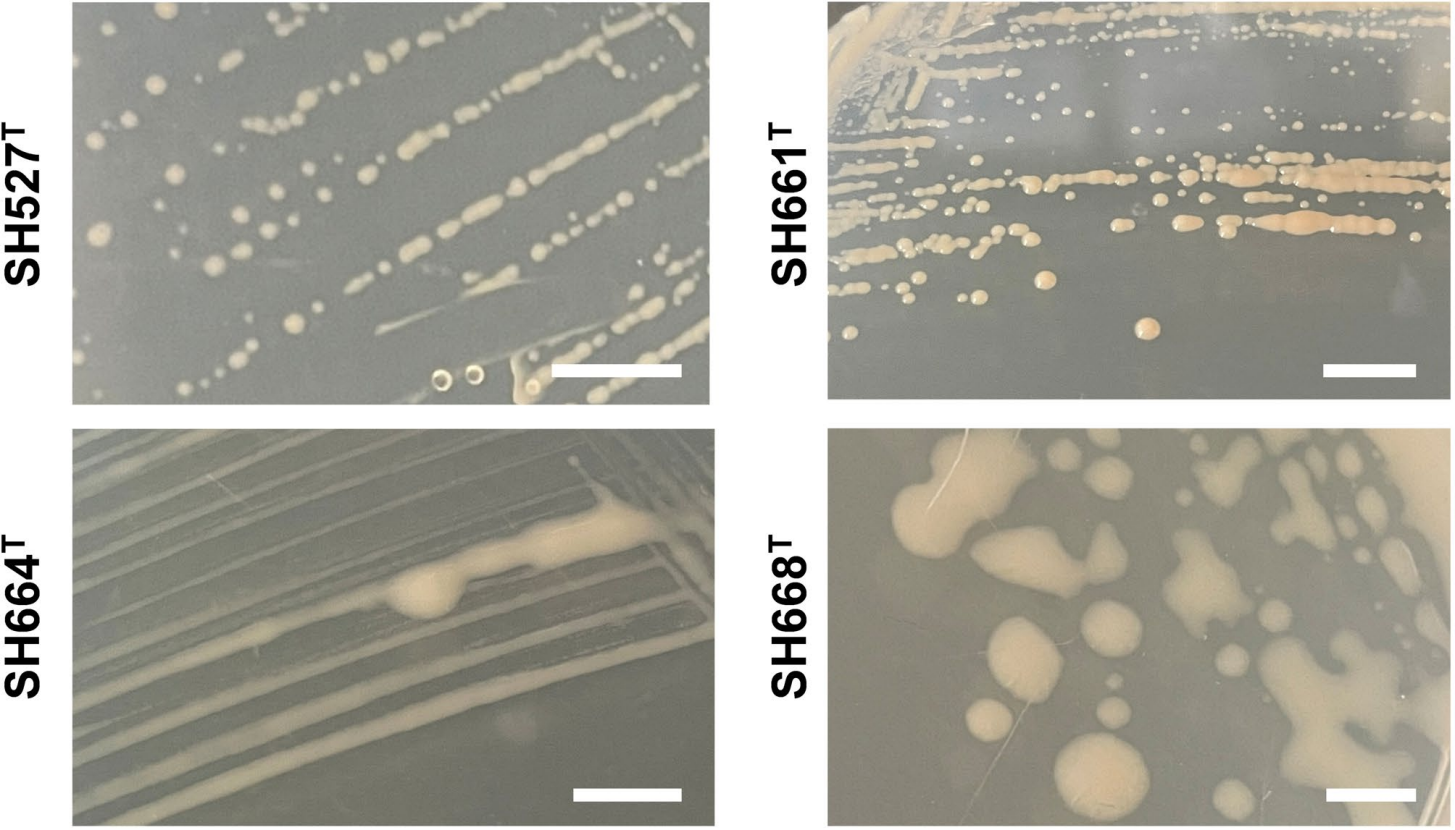
Appearance of colonies on plates. Colonies of the four strains have the same color, but colonies differ in size, shape and consistency. Strains SH527^T^ and SH661^T^ form smaller circular colonies, whereas colonies of the strains SH664^T^ and SH668^T^ are much bigger, irregular and have a slimier consistency. The scale bars represent 0.5 cm.

**Fig. 5:**
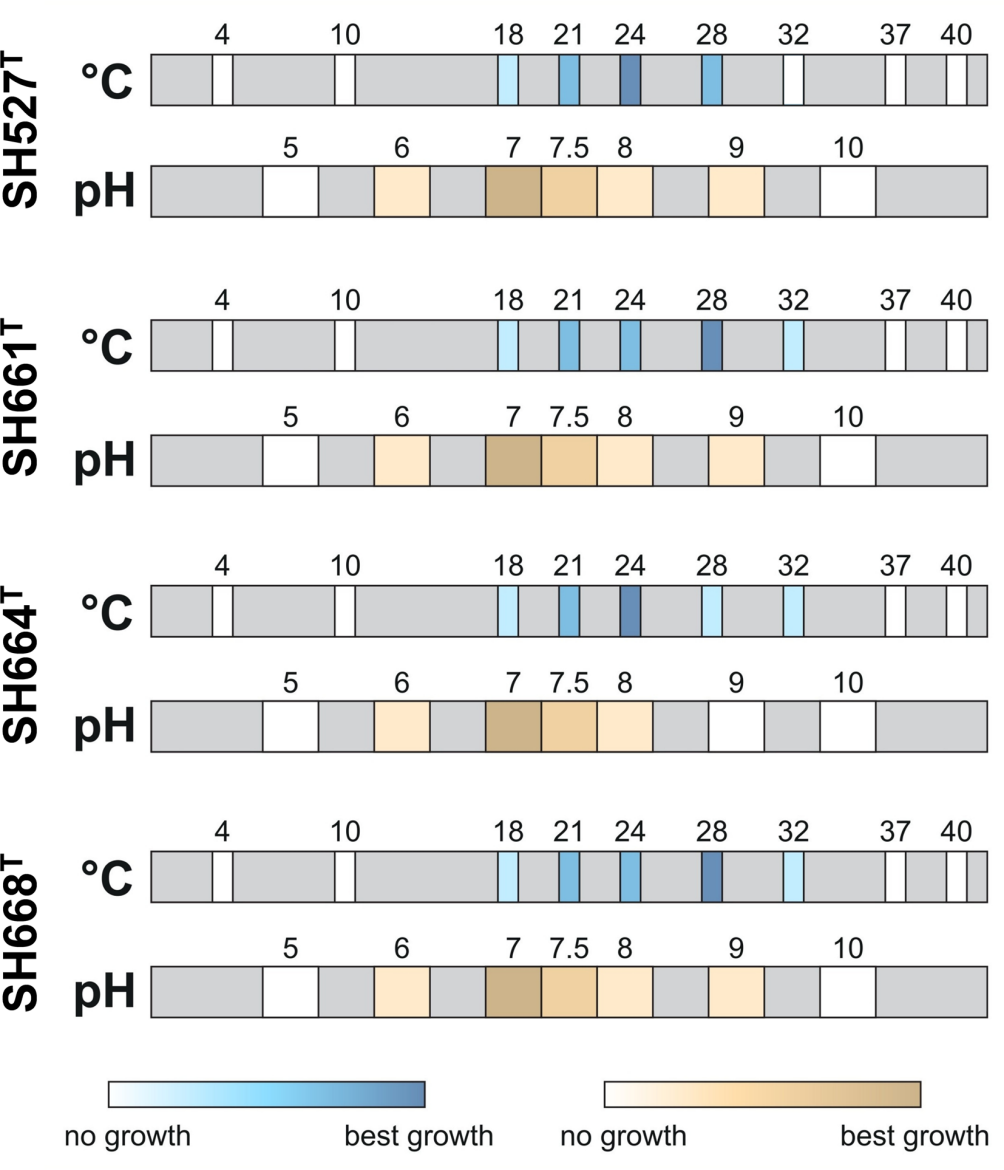
Determination of pH and temperature optima. The optimal growth conditions were determined by growth experiments on various temperatures and pH values. Squares labelled with numbers indicate tested temperature or pH conditions while the intensity of the color indicates the amount of growth observed.

On agar plates and in liquid culture, all four strains are non-pigmented and show an off-white ivory color (Fig. 4) similar to that of the type strain of *P. piriforme*. Strains SH527^T^ and SH661^T^ form round and convex colonies with entire margins and low to moderate diameters. Colonies of strains SH664^T^ and SH668^T^ are much larger, round or irregular and with a slimier consistency (Fig. 4). All four strains show growth at 18–30 or 32 °C with optimal growth at either 24 or 28 °C and prefer pH-neutral conditions (Fig. 5; Table 2). Extracellular pH values between 6.0 and 9.0 still allow for biomass formation. In contrast, *P. piriforme* P3^T^ shows optimal growth under slightly acidic conditions but in the same temperature range (24–28 °C) as the here described isolates. The aerobic heterotrophic lifestyle of *P. piriforme* P3^T^ is also followed by the four novel strains (Table 2).

**Table 2:** Comparison of phenotypic characteristics. Data on *P. piriforme* P3^T^ was obtained from the species description^41^.

Characteristics	*P*. piriforme P3^T^	SH527^T^	SH661^T^	SH664^T^	SH668^T^
Sampling information					
Location	Valaam Island, Northern Russia	Strohbrück, Germany	Strohbrück, Germany	Strohbrück, Germany	Strohbrück, Germany
Sampled material	Littoral wetland of a boreal lake	Sewage treatment sludge	Leakage water from industrial compost heap	Leakage water from industrial compost heap	Leakage water from industrial compost heap
Phenotypic features					
Pigmentation	Non-pigmented	Non-pigmented	Non-pigmented	Non-pigmented	Non-pigmented
Cell Shape	Ellipsoidal to pear-shaped	Ellipsoidal to pear-shaped	Ellipsoidal to pear-shaped	Ellipsoidal to pear-shaped	Ellipsoidal to pear-shaped
Size (Length x width) (µm)	1.7–2.8 x 0.9–1.3	1.9 ± 0.3 x 1.4 ± 0.2	1.8 ± 0.2 x 1.3 ± 0.1	1.6 ± 0.1 x 1.2 ± 0.1	1.8 ± 0.2 x 1.3 ± 0.2
Cell division mode	Asymmetric (polar budding)	Asymmetric (polar budding)	Asymmetric (polar budding)	Asymmetric (polar budding)	Asymmetric (polar budding)
Temperature range (optimum) (°C)	10–30 (20–28)	18–30 (24)	18–32 (28)	18–32 (24)	18–32 (28)
pH Range (optimum)	4.2–7.1 (6.0–6.5)	6.0–9.0 (7.0)	6.0–9.0 (7.0)	6.0–8.0 (7.0)	6.0–9.0 (7.0)
Relation to oxygen	Aerobic	Aerobic	Aerobic	Aerobic	Aerobic
Motility	Yes, daugther cells	Yes, daugther cells	Yes, daugther cells	Yes, daugther cells	Yes, daugther cells
Stalks	Yes	n.o.	n.o.	n.o.	n.o.
Aggregates	Yes, rosettes	No	No	No	No

n.o. not observed

**Fig. 6:**
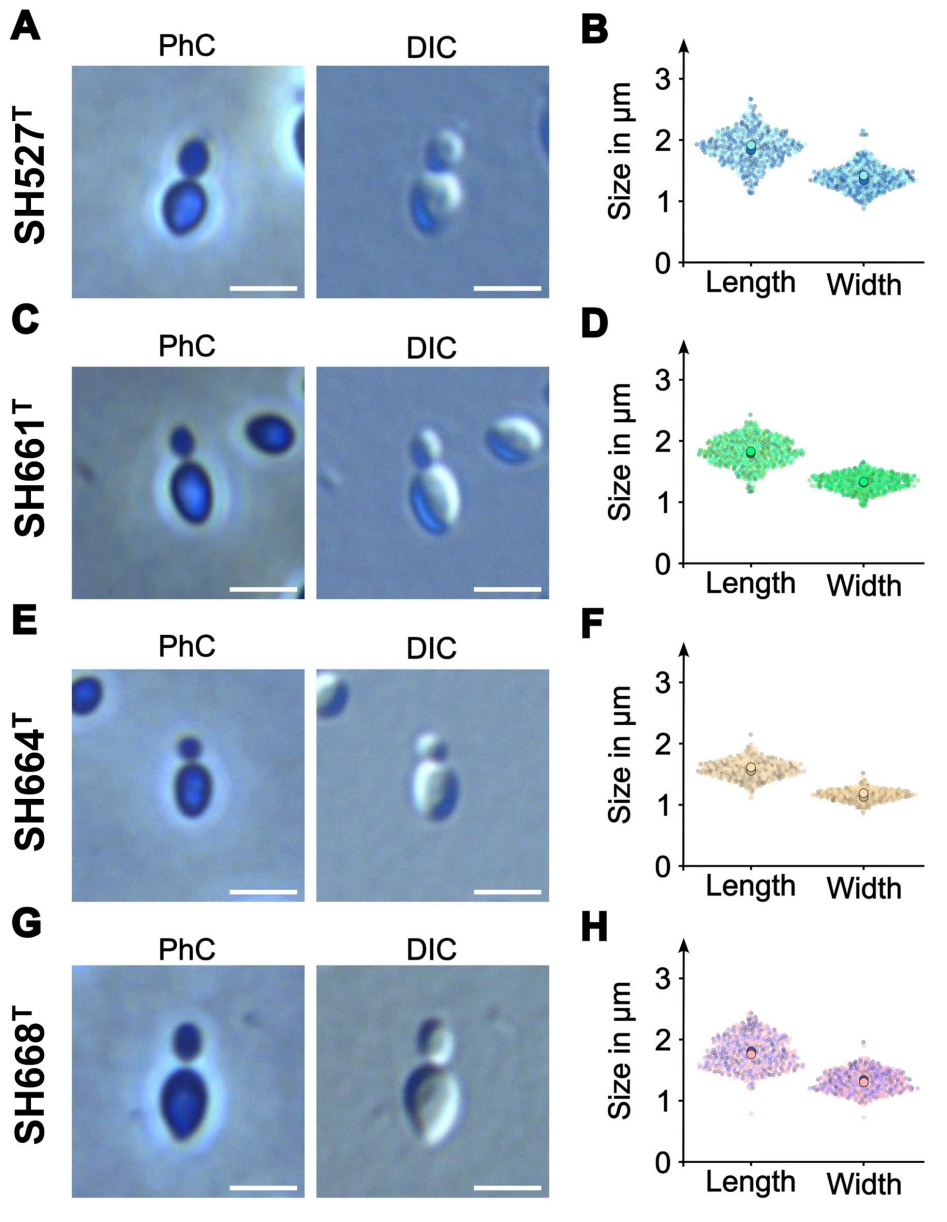
Cell morphology and cell sizes. Phase contrast (PhC) and differential interference contrast (DIC) images of dividing cells. Smaller daughter cells emerging from larger mother cells are depicted in the early division stage. Cell sizes were determined from three biological replicates. Larger circles indicate mean values of each replicate. The scale bars represent 2 μm.

On the microscopic scale, cells of all four strains appear ellipsoid to pear-shaped and display comparable cell dimensions when compared against each other (Fig. 6). The cells are slightly more roundish compared to strain P3^T^; however, it cannot be excluded that this is an effect of the different cultivation media used. Microscopic aggregates could not be observed for the four isolates. Like other members of the family *Planctomycetaceae*, cells divide asymmetrically through a polar budding process, in which a small daughter cell emerges one at a time on one pole of the larger mother cell. In all four strains, the daughter cell forms at the pole which is rounder and less pointy. The daughter cells then grow over time and eventually pinch off from the mother cell (Fig. 6). Stalks were not visible during light microcopy.

## Conclusion

The four novel isolates show clear distinction when compared against each other, but also when compared against the currently sole characterized member of the genus *Planctomicrobium*. Thus, the data supports a position for the novel strains within the genus *Planctomicrobium* but individually delineated from the sole species *P. piriforme*. Hence, we introduce four novel species, represented by the four presented isolates as respective type strains.

### Description of *Planctomicrobium limosum *sp. nov.

li.mo’sum. L. neut. adj. *limosum*, muddy; pertaining to sludge, from which the type strain was isolated.

Cells are ellipsoidal to pear-shaped with an average length and width of 1.9 × 1.4 μm. Colonies are non-pigmented and have an off-white ivory color. Cells form small round and convex colonies on agar plates. Daughter cells are motile. Limnic and aerobic heterotroph with a mesophilic and neutrophilic growth profile. The type strain is SH527^T^ (= CECT 30937^T^ = KCTC 102136^T^). It was isolated from sewage sludge in Northern Germany. The type strain grows over a range of 18–30 °C and pH 6.0–9.0 with optimal growth at 24 °C and pH 7.0. It has a genome size of 5.49 Mbp with a DNA G + C content of 52.9% and lacks plasmids.

### Description of *Planctomicrobium stercoris* sp. nov.

ster’co.ris. L. gen. n. *stercoris*, of compost; from which the strain was isolated.

Cells are ellipsoidal to pear-shaped with an average length and width of 1.8 × 1.3 μm. Colonies are non-pigmented and have an off-white ivory color. Cells form round and convex colonies with moderate sizes on agar plates. Daughter cells are motile. Limnic and aerobic heterotroph with a mesophilic and neutrophilic growth profile. The type strain is SH661^T^ (= DSM 116760^T^ = KCTC 102128^T^). It was isolated from leakage water of an industrial compost heap in Northern Germany. The type strain grows over a range of 18–32 °C and pH 6.0–9.0 with optimal growth at 28 °C and pH 7.0. It has a genome size of 5.86 Mbp with a DNA G + C content of 57.2% and lacks plasmids.

### Description of *Planctomicrobium aquicomposti* sp. nov.

a.qui.com.pos’ti. L. fem. n. *aqua*, water; N.L. neut. n. *compostum*, compost; N.L. gen. n. *aquicomposti*, pertaining to the isolation from leakage water of a compost heap.

Cells are ellipsoidal to pear-shaped with an average length and width of 1.6 × 1.2 μm. Colonies are non-pigmented and have an off-white ivory color. Cells form larger circular or irregular colonies with a slimy consistency on agar plates. Daughter cells are motile. Limnic and aerobic heterotroph with a mesophilic and neutrophilic growth profile. The type strain is SH664^T^ (= DSM 116388^T^ = KCTC 102081^T^). It was isolated from leakage water of an industrial compost heap in Northern Germany. The type strain grows over a range of 18–32 °C and pH 6.0–8.0 with optimal growth at 24 °C and pH 7.0. It has a genome size of 5.22 Mbp with a DNA G + C content of 59.2% and lacks plasmids.

### Description of *Planctomicrobium mucosum* sp. nov.

mu.co’sum. L. neut. adj. *mucosum*, slimy, mucous; based on the slimy appearance of the colonies of the type strain.

Cells are ellipsoidal to pear-shaped with an average length and width of 1.8 × 1.3 μm. Colonies are non-pigmented and have an off-white ivory color. Cells form large circular or irregular colonies with a slimy consistency on agar plates. Daughter cells are motile. Limnic and aerobic heterotroph with a mesophilic and neutrophilic growth profile. The type strain is SH668^T^ (= DSM 116585^T^ = KCTC 102080^T^). It was isolated from leakage water of an industrial compost heap in Northern Germany. The type strain grows over a range of 18–32 °C and pH 6.0–9.0 with optimal growth at 28 °C and pH 7.0. It has a genome size of 4.81 Mbp with a DNA G + C content of 52.7% and lacks plasmids.

### Emended description of the genus *Planctomicrobium* Kulichevskaya et al. 2015

Characteristics of the genus are as previously described^41^ with the following modifications: Colonies have an off-white ivory color and can vary in size. Neutrophilic or mildly acidophilic. The DNA G + C content falls in the range of 52–60%.

## Data Availability

The 16S rRNA gene sequences of the novel isolates were deposited in the GenBank database under the following accession numbers: PV904163 (SH527^T^), PV904164 (SH661^T^), PV904179 (SH664^T^) and PV904181 (SH668^T^). Genome sequence information is available from NCBI under the accession numbers CP195790 (SH527^T^), CP195791 (SH661^T^), CP195792 (SH664T) and CP195793 (SH668^T^).
